# Diversity of *Bacillus* Isolates from the Sake Brewing Process at a Sake Brewery

**DOI:** 10.3390/microorganisms9081760

**Published:** 2021-08-18

**Authors:** Emi Kanamoto, Keigo Terashima, Yoshiji Shiraki, Hiromi Nishida

**Affiliations:** 1 Department of Biotechnology, Toyama Prefectural University, 5180 Kurokawa, Imizu, Toyama 939-0398, Japan; t816015@st.pu-toyama.ac.jp; 2 Terashima Sake Store, 1-4-1 Midoricho, Toyama, Toyama 930-0038, Japan; terashima@tokinoya.jp; 3 Shiraki Tsunesuke Sake Brewery, 61 Kadoyakado, Gifu, Gifu 501-2528, Japan; koshu@daruma-masamune.co.jp

**Keywords:** *Bacillus*, bacteria, environment, ethanol tolerance, *kuratsuki*, PCR, sake brewery

## Abstract

We collected 92 isolates belonging to the genus *Bacillus* from the sake brewing process at Shiraki Tsunesuke Sake Brewery in Gifu, Japan to determine whether there is strain specificity at individual sake breweries. After distributing the isolates into seven groups, we observed that at least two groups (68 isolates) were *kuratsuki* bacteria at Shiraki Tsunesuke Sake Brewery. The *kuratsuki* *Bacillus* isolates were collected from different samples at the early and late stages of sake brewing in 2021 and 2019, respectively. These results showed that *kuratsuki* *Bacillus* entered the sake brewing process at this location. These *kuratsuki Bacillus* isolates had a high ethanol tolerance. Our previous paper showed the existence of *kuratsuki Kocuria* at Narimasa Sake Brewery in Toyama, Japan, but this study demonstrated that it is not found at Shiraki Tsunesuke Sake Brewery. Therefore, each sake brewery has specific *kuratsuki* bacterial strains, which are isolated with high frequency and contribute a specific flavor or taste to each sake brewery.

## 1. Introduction

**Figure 1:**
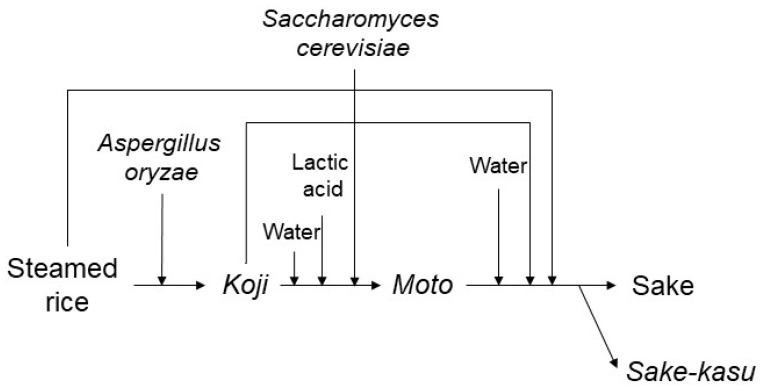
Sake production scheme. *Koji* is a mixture of steamed rice and *Aspergillus oryzae*. *Moto* is an ethanol fermentation starter. Sake and *sake-kasu* are separated by filtration.

Sake is a traditional Japanese alcoholic drink, which has been brewed for over 1300 years [1]. At present, ~1400 sake breweries are widely distributed throughout Japan. *Aspergillus oryzae* is a fungus that converts rice starch into sugars, and then another fungus (yeast), *Saccharomyces cerevisiae*, converts the sugars into ethanol during sake brewing (Figure 1). Sake brewing is not performed aseptically, so bacterial flora analysis based on DNA sequence comparison in sake can detect several bacterial DNA [2,3,4,5,6], indicating that the bacteria may enter the production process by chance and briefly grow. The research question was whether all bacteria entered by chance or not. Thus, are there microorganisms that inevitably enter during the sake production process?

Lactic acid treatment is performed before the addition of *S. cerevisiae* for the effective ethanol fermentation (Figure 1). *Kuratsuki* (living in the sake brewery) lactic acid bacteria have been used for preventing the growth of other bacteria, which can lead to sake spoilage [3]. In the traditional method of producing fermentation starter, the *kuratsuki* lactic acid bacteria grow and produce lactic acid [3]. The lactic acid bacteria interact with other microorganisms in sake [7,8], but are killed by self-produced lactic acid or ethanol produced by *S. cerevisiae*. In contrast, some highly ethanol-tolerant lactic acid bacteria, for example *Lactobacillus fructivorans*, spoil sake [9,10,11], demonstrating that lactic acid bacteria can have a positive and a negative effect on sake brewing.

To elucidate whether *kuratsuki* bacteria other than lactic acid bacteria exist during the sake brewing process, 46 bacterial isolates were collected at the early stage of the sake brewing process from Narimasa Sake Brewery in Toyama, Japan [6,12]. Among the isolates, 23 had similar sequences to the genera *Kocuria*, 12 to *Staphylococcus*, 6 to *Bacillus*, 2 to *Leifsonia*, 2 to *Microbacterium*, and 1 to *Enterococcus*, inferred from the partial 16S rRNA gene sequences [6,12]. All isolates were not lactic acid bacteria. We showed that the *Kocuria* isolates have inhabited Narimasa Sake Brewery and are *kuratsuki* bacteria [12,13]. *Kocuria* does not belong to the phylum Firmicutes but Actinobacteria [14]. This was the first report of actinomycete living at a sake brewery. Recently, we showed that the *kuratsuki* bacteria in the Narimasa Sake Brewery interact with *S. cerevisiae* and affect flavor of the sake products [15]. The bacterial flora affects the chemical components in sake [16,17].

Based on the DNA sequence data in sake, *Kocuria* is specific to Narimasa Sake Brewery [6]. However, it was uncertain whether *Kocuria* is not isolated from other sake breweries, so we elucidated the same in this study. In 2019, six bacterial isolates were collected from the sample at the late stage of the sake brewing process of Shiraki Tsunesuke Sake Brewery in Gifu, Japan. In this study, we isolated bacteria during the early stage of the sake brewing processes of this sake brewery, compared them, and then discussed them.

## 2. Materials and Methods

### 2.1. Bacterial Isolation

Bacterial isolation was performed for four different sampling date samples from Shiraki Tsunesuke Sake Brewery (Table 1). Three different media, Difco Marine Broth 2216 (DMB) (BD, Franklin Lakes, NJ, USA), LB (10 g/L of tryptone, 10 g/L of NaCl, 5 g/L of yeast extract), and TGY (5 g/L of tryptone, 3 g/L of yeast extract, 1 g/L of glucose), containing 25 μg/mL of cycloheximide, were used [6]. Each sample (30 μL) was added to each medium (20 mL) and cultured at 15 °C and 30 °C for 72 h. Sake brewing was performed at 15 °C. Then, a single colony was chosen on the TGY agar plate.

### 2.2. Colony PCR

The V3–V4 region of the 16S rRNA gene from each single colony was amplified using region-specific PCR primers [12], designed by modification of a method by Illumina, San Diego, USA. The PCR products were sequenced using the primer 5′-TGTATAAGAGACAGGACTAC-3′.

### 2.3. Phylogenetic Analysis

Multiple alignment (Supplementary File S1) was constructed using MEGA X [18] for 92 DNA sequences of the bacterial isolates from Shiraki Tsunesuke Sake Brewery, 6 DNA sequences of the *Bacillus* isolates from Narimasa Sake Brewery [6,12], and 10 DNA sequences of the known *Bacillus* species from DNA database (Table 2). A neighbor-joining tree was constructed with 1000 bootstrap replicates using MEGA X [18]. The evolutionary distances were computed using the maximum composite likelihood method [19], and are in the units of the number of base substitutions per site. All positions containing gaps and missing data were eliminated (complete deletion option). There were a total of 392 positions in the final dataset.

### 2.4. Ethanol Resistance Test

The isolates a12 and C3 were statically cultured in TGY including 20% ethanol at 30 °C. Each sample (100 μL) was spread on a TGY agar to count the number of colonies.

### 2.5. Statistical Test

A chi-square test was performed between isolated numbers from the cultures at 15 °C and 30 °C using the software R (http://www.R-project.org/).

## 3. Results

### 3.1. All Isolates from Shiraki Tsunesuke Sake Brewery belong to the Genus Bacillus

We obtained 6 DNA sequences from the samples at the late stage of the sake brewing process in 2019, 28 at the early stage on 26 January 2021, 30 at the early stage on 28 January 2021, and 28 at the early stage on 9 February 2021 (Table 1). Using the basic local alignment search on National Center for Biotechnology Information (https://www.ncbi.nlm.nih.gov/), all 92 DNA sequences of bacterial isolates from Shiraki Tsunesuke Sake Brewery were found to be DNA sequences of the genus *Bacillus*. All partial 16S rRNA gene sequences are shown in Supplementary File S1.

### 3.2. Bacillus Isolates Distributed to Seven Different Groups

**Figure 2:**
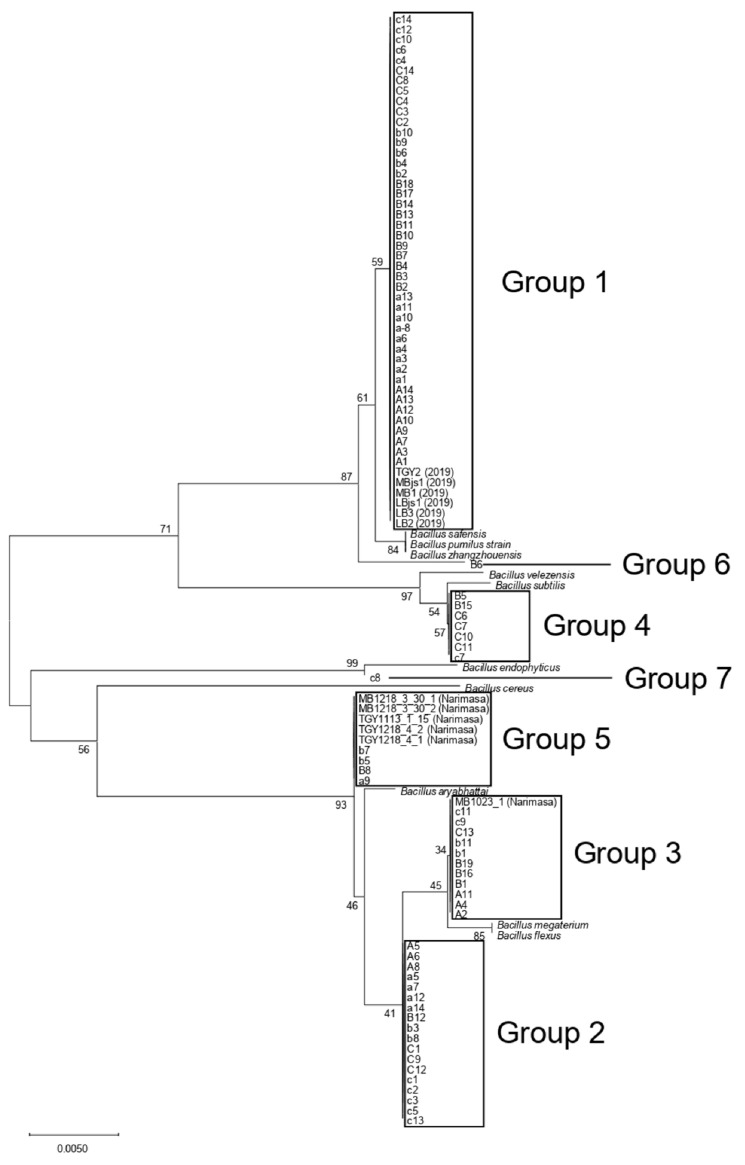
Phylogenetic relationships among 16S rRNA gene sequences of the 92 isolates from Shiraki Tsunesuke Sake Brewery, 6 isolates from Narimasa Sake Brewery, and 10 known species of *Bacillus*. A neighbor-joining tree was constructed with 1000 bootstrap replicates using MEGA X [18]. The evolutionary distances were computed using the maximum composite likelihood method [19], and are in the units of the number of base substitutions per site. All positions containing gaps and missing data were eliminated (complete deletion option). There were total 392 positions in the final dataset.

**Figure 3:**
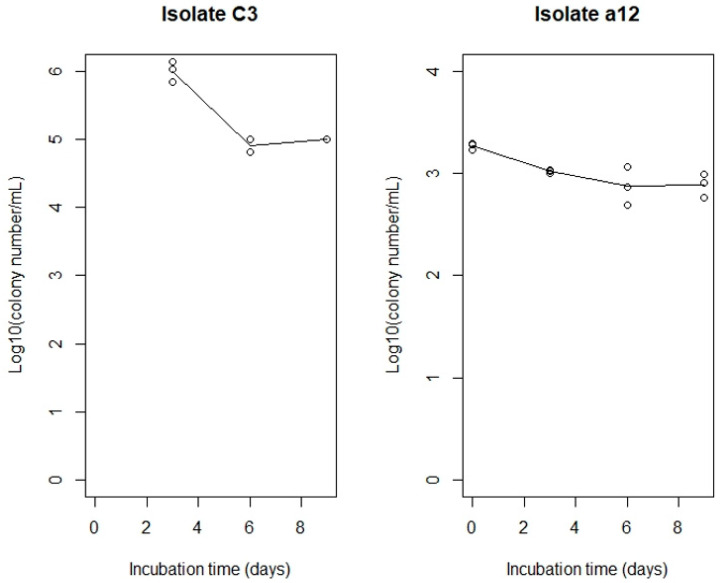
Growth of *kuratsuki Bacillus* isolates in TGY including 20% ethanol. The isolates C3 and a12 belonging to Groups 1 and 2, respectively, were statically cultured in TGY including 20% ethanol at 30 °C.

Phylogenetic analysis showed that the bacterial DNA sequences from Shiraki Tsunesuke Sake Brewery could be distributed into seven groups (Figure 2). The groups were numbered 1–7, with Group 1 being the largest and Groups 6 and 7 being the smallest. Group 1 consisted of 50 sequences, including all six DNA sequences from the samples in 2019 (Figure 2). This group consisted of DNA sequences from three different samples at the early stage of the sake brewing process and contained no sequences from Narimasa Sake Brewery. Thus, this largest group was specific to Shiraki Tsunesuke Sake Brewery. Group 2 consisted of 18 sequences from three different samples, with no sequences from Narimasa Sake Brewery (Figure 2). The isolates of Groups 1 and 2 were obtained from both cultures at 15 °C and 30 °C (Table 3). The isolation frequency bias was not detected (*p* > 0.05, chi-square test) between different sampling dates of cultures at 15 °C and 30 °C in each group and between different groups of cultures at 15 °C and 30 °C. An ethanol resistance test showed that isolate C1 belonging to Group 1 and isolate a12 belonging to Group 2 had an ethanol resistance to 20% ethanol (Figure 3).

Group 3 consisted of 11 sequences from three different samples (Figure 2). This group included one sequence from Narimasa Sake Brewery, indicating that it was not specific to Shiraki Tsunesuke Sake Brewery. Group 4 consisted of seven sequences from two different samples, with no sequences from Narimasa Sake Brewery (Figure 2). Group 5 consisted of four sequences from two different samples (Figure 2). This group included five sequences from Narimasa Sake Brewery, indicating that this group was not specific to Shiraki Tsunesuke Sake Brewery. In addition, Groups 6 and 7 were located alone (Figure 2).

Group 1 clustered with *B. safensis*, *B. pumilus*, *B. zhangzhouensis*, and isolate B6 (Group 6) with 87% support of bootstrap (Figure 2). Groups 2, 3, and 5 were clustered with *B. aryabhattai*, *B. flexus*, and *B. megaterium* with 93% support of bootstrap (Figure 2). Group 4 was clustered with *B. subtilis* and *B. velezensis* with 97% support of bootstrap (Figure 2). Group 7 was clustered with *B. endophyticus* with 99% support of bootstrap (Figure 2).

## 4. Discussion

Although *Bacillus* is frequently detected in the sake brewery environments [2], it was surprising that all 92 isolates from four different samples at Shiraki Tsunesuke Sake Brewery belong to the genus *Bacillus*. In Narimasa Sake Brewery, 46 isolates from 6 different samples belong to the 6 different genera: *Kocuria* (23 isolates), *Staphylococcus* (12 isolates), *Bacillus* (6 isolates), *Leifsonia* (2 isolates), *Microbacterium* (2 isolates), and *Enterococcus* (1 isolate) [6,12]. This strongly suggests that different sake breweries have different bacteria compositions entering the brewing process. Narimasa Sake Brewery has *kuratsuki Kocuria* at the different species level [6,12,13], leading us to hypothesize that Shiraki Tsunesuke Sake Brewery may also have *kuratsuki Bacillus* at the different species level. *Bacillus* species have both positive and negative effects on drinks and foods [20,21,22,23,24].

We believe that the *Bacillus* isolates of Groups 1 and 2 are *kuratsuiki* bacteria in Shiraki Tsunesuke Sake Brewery because these *Bacillus* isolates are specific to this sake brewery. In particular, the isolates of Group 1 are *kuratsuki* bacteria because this group includes the *Bacillus* strains that were isolated in 2019 at late sake production process, which has a high ethanol concentration (>15% of ethanol). Considering that the same *Bacillus* isolates were collected from the different samples with different sampling year, these isolates may have inhabited the Shiraki Tsunesuke Sake Brewery and entered the annual sake brewing process. The *Bacillus* isolates of Group 1 have a high ethanol tolerance and survive at the late stage of the sake brewing process. In contrast, Group 2 does not include the *Bacillus* isolates in 2019. This suggested that the *Bacillus* isolates of Group 2 might be less ethanol-tolerant compared with those of Group 1. However, the isolates of Group 2 had a high ethanol tolerance (Figure 3).

Group 3 consists of isolates from different three samples as well as Groups 1 and 2 (Table 3). However, Group 3 includes the isolate MB1023_1 from Narimasa Sake Brewery (Figure 1), indicating that this group is not specific to Shiraki Tsunesuke Sake Brewery. It is possible that different sake breweries have common *kuratsuki* bacteria. However, *kuratsuki* bacteria undergo genome modification during the sake brewing to adapt to the brewery environments [12]. For example, although the optimal growth temperature of the genus *Kocuria* is generally 30–37 °C, the *kuratsuki Kocuria* isolates in Narimasa Sake Brewery is 15 °C, which is the same temperature during the sake brewing process [12]. Even if the two different sake breweries have a common *kuratsuki* bacterial genus *Bacillus*, those genomes should differ at the species or strain level. To elucidate it, the genome sequence analysis is necessary.

Groups 4–7 do not consist of isolates from all of the different three samples (Table 3). In addition, from the viewpoint of detection frequency, the detection frequency of isolates in these groups is so low that these isolates have a low possibility of *kuratsuki* bacteria in Shiraki Tsunesuke Sake Brewery. Because *kuratsuki* bacteria have inhabited a sake brewery for a long time and entered every sake brewing process, the detection frequency of *kuratsuki* bacteria is usually higher than that of other bacteria entered in the sake brewing process.

Groups 3 and 5 consist of isolates from both Narimasa and Shiraki Tsunesuke breweries. It is uncertain if these isolates are also *kuratsuki Bacillus*. Nevertheless, these isolates may have an affinity to sake brewing. Thus, these *Bacillus* isolates may influence metabolism of *S. cerevisiae* and/or other microorganisms. If so, both *kuratsuki* bacteria and other bacteria may affect flavor and taste specific to each sake brewery. To elucidate how the *Bacillus* isolates affect chemical components in sake, further research is warranted.

**Figure 4:**
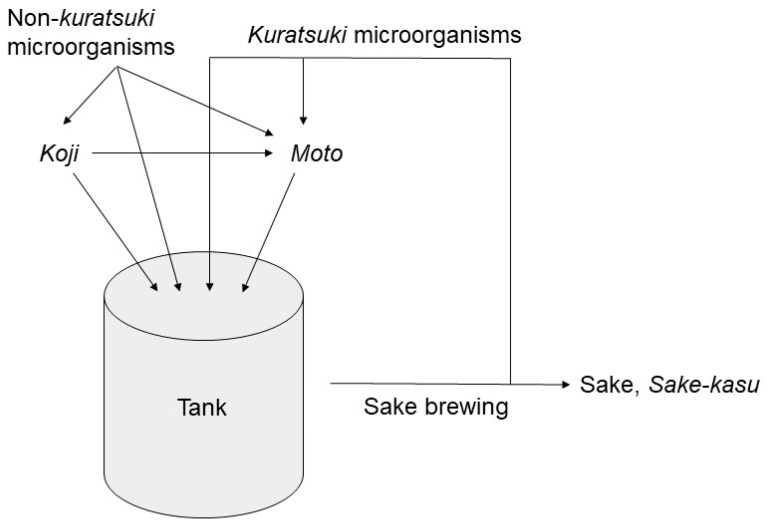
Difference between *kuratsuki* and non-*kuratsuki* microorganisms in the sake brewing process. *Koji* contains *Aspergillus oryzae*. *Moto* contains *A. oryzae* and *Saccharomyces cerevisiae*. *S. cerevisiae* in *moto* produces ethanol during the sake brewing process.

This study shows that different sake breweries have distinct *kuratsuki* bacteria; Narimasa Sake Brewery has *kuratsuki Kocuria* and Shiraki Tsunesuke Sake Brewery has *kuratsuki Bacillus*. The genera *Bacillus* and *Kocuria* differ phylogenetically, and these different bacteria are specific to each sake brewery, having evolved in each brewery. This means that *kuratsuki* bacteria differ from other environmental microorganisms (Figure 4), in that they have a life cycle in the sake brewery and enter the sake brewing process. In contrast, environmental microorganisms enter the sake brewing process by chance. We have a hypothesis that *kuratsuki* bacteria were derived from the environmental microorganisms and they have adapted to the sake brewing conditions by continuing to enter the sake production process. *Kuratsuki* bacteria may have a key role in the interaction among microorganisms during the sake production process [13].

It is important that *kuratsuki* bacteria do not consist of single lineage but multiple lineages. This strongly suggests that *kuratsuki* bacteria evolved in sake tank during the sake brewing (Figure 4) and then the bacteria distributed into the environment of the sake brewery. The *kuratsuki* bacteria have continued to enter and distribute repeatedly for a long time. In each sake brewing, *kuratsuki* bacteria may be selected from the multiple lineages. Considering the evolution of *kuratsuki* bacteria, their genome analyses are important. In *kuratsuki Kocuria* isolates, plasmid and transposon were identified and genome modification between the different isolates were suggested [12]. *Kuratsuki* yeasts also probably have multiple lineages. However, at present, most sake breweries do not use *kuratsuki* yeasts but selected sake yeasts (*Kyokai* yeasts) that are managed by the Brewery Society of Japan. Each *Kyokai* yeast consists of a single lineage. It is possible that some *Kyokai* yeasts are not suitable for some sake brewery environments.

## 5. Conclusions

Microorganisms play an important role in the production of flavor and taste, and influence the sake quality. Although bacteria die at the final stage of sake production, bacteria in sake are alive and grow temporarily during the sake production process. *Kuratsuki* bacteria are not any contaminated bacteria, which are always entered in sake brewing. To our knowledge, although research on *kuratsuki* yeasts has been carried out, research on *kuratsuki* bacteria has not been performed except for lactic acid bacteria. We are changing the characteristics of sake and will produce a unique sake with different tastes and flavors by exchanging the *kuratsuki* bacteria from different sake breweries during the production process.

## Figures and Tables

**Table 1:** *Bacillus* isolates from the sake brewing process in Shiraki Tsunesuke Sake Brewery.

Isolate	Sample Number	Sampling Date	Process Stage	Medium
LB2	Sample 1	30 January 2019	Late	LB
LB3	Sample 1	30 January 2019	Late	LB
LBjs1	Sample 1	30 January 2019	Late	LB
MB1	Sample 1	30 January 2019	Late	DMB
MBjs1	Sample 1	30 January 2019	Late	DMB
TGY2	Sample 1	30 January 2019	Late	TGY
A1	Sample 2	26 January 2021	Early	DMB
A2	Sample 2	26 January 2021	Early	DMB
A3	Sample 2	26 January 2021	Early	LB
A4	Sample 2	26 January 2021	Early	LB
A5	Sample 2	26 January 2021	Early	LB
A6	Sample 2	26 January 2021	Early	TGY
A7	Sample 2	26 January 2021	Early	TGY
A8	Sample 2	26 January 2021	Early	TGY
A9	Sample 2	26 January 2021	Early	DMB
A10	Sample 2	26 January 2021	Early	TGY
A11	Sample 2	26 January 2021	Early	TGY
A12	Sample 2	26 January 2021	Early	TGY
A13	Sample 2	26 January 2021	Early	LB
A14	Sample 2	26 January 2021	Early	LB
a1	Sample 2	26 January 2021	Early	LB
a2	Sample 2	26 January 2021	Early	LB
a3	Sample 2	26 January 2021	Early	LB
a4	Sample 2	26 January 2021	Early	LB
a5	Sample 2	26 January 2021	Early	LB
a6	Sample 2	26 January 2021	Early	DMB
a7	Sample 2	26 January 2021	Early	DMB
a8	Sample 2	26 January 2021	Early	DMB
a9	Sample 2	26 January 2021	Early	DMB
a10	Sample 2	26 January 2021	Early	DMB
a11	Sample 2	26 January 2021	Early	TGY
a12	Sample 2	26 January 2021	Early	TGY
a13	Sample 2	26 January 2021	Early	TGY
a14	Sample 2	26 January 2021	Early	TGY
B1	Sample 3	28 January 2021	Early	LB
B2	Sample 3	28 January 2021	Early	LB
B3	Sample 3	28 January 2021	Early	LB
B4	Sample 3	28 January 2021	Early	LB
B5	Sample 3	28 January 2021	Early	LB
B6	Sample 3	28 January 2021	Early	LB
B7	Sample 3	28 January 2021	Early	LB
B8	Sample 3	28 January 2021	Early	TGY
B9	Sample 3	28 January 2021	Early	TGY
B10	Sample 3	28 January 2021	Early	TGY
B11	Sample 3	28 January 2021	Early	TGY
B12	Sample 3	28 January 2021	Early	TGY
B13	Sample 3	28 January 2021	Early	TGY
B14	Sample 3	28 January 2021	Early	DMB
B15	Sample 3	28 January 2021	Early	DMB
B16	Sample 3	28 January 2021	Early	DMB
B17	Sample 3	28 January 2021	Early	DMB
B18	Sample 3	28 January 2021	Early	DMB
B19	Sample 3	28 January 2021	Early	DMB
b1	Sample 3	28 January 2021	Early	LB
b2	Sample 3	28 January 2021	Early	LB
b3	Sample 3	28 January 2021	Early	LB
b4	Sample 3	28 January 2021	Early	DMB
b5	Sample 3	28 January 2021	Early	DMB
b6	Sample 3	28 January 2021	Early	DMB
b7	Sample 3	28 January 2021	Early	DMB
b8	Sample 3	28 January 2021	Early	TGY
b9	Sample 3	28 January 2021	Early	TGY
b10	Sample 3	28 January 2021	Early	TGY
b11	Sample 3	28 January 2021	Early	TGY
C1	Sample 4	9 February 2021	Early	LB
C2	Sample 4	9 February 2021	Early	LB
C3	Sample 4	9 February 2021	Early	LB
C4	Sample 4	9 February 2021	Early	LB
C5	Sample 4	9 February 2021	Early	DMB
C6	Sample 4	9 February 2021	Early	DMB
C7	Sample 4	9 February 2021	Early	DMB
C8	Sample 4	9 February 2021	Early	DMB
C9	Sample 4	9 February 2021	Early	TGY
C10	Sample 4	9 February 2021	Early	TGY
C11	Sample 4	9 February 2021	Early	TGY
C12	Sample 4	9 February 2021	Early	TGY
C13	Sample 4	9 February 2021	Early	TGY
C14	Sample 4	9 February 2021	Early	TGY
c1	Sample 4	9 February 2021	Early	LB
c2	Sample 4	9 February 2021	Early	LB
c3	Sample 4	9 February 2021	Early	LB
c4	Sample 4	9 February 2021	Early	LB
c5	Sample 4	9 February 2021	Early	DMB
c6	Sample 4	9 February 2021	Early	DMB
c7	Sample 4	9 February 2021	Early	DMB
c8	Sample 4	9 February 2021	Early	DMB
c9	Sample 4	9 February 2021	Early	DMB
c10	Sample 4	9 February 2021	Early	DMB
c11	Sample 4	9 February 2021	Early	TGY
c12	Sample 4	9 February 2021	Early	TGY
c13	Sample 4	9 February 2021	Early	TGY
c14	Sample 4	9 February 2021	Early	TGY

**Table 2:** *Bacillus* species used in the 16S rRNA sequence comparison.

Species	Strain	Sequence ID
*Bacillus aryabhattai*	B8W22	NR_115953.1
*B. cereus*	ATCC 14579	NR_074540.1
*B. endophyticus*	2DT	MK554520.1
*B. flexus*	IFO 15715	NR_024691.1
*B. megaterium*	ATCC 14581	AB271751.1
*B. pumilus*	ATCC 7061	NR_043242.1
*B. safensis*	FO-36b	NR_041794.1
*B. subtilis*	IAM 12118	NR_112116.1
*B. velezensis*	FZB42	NR_075005.2
*B. zhangzhouensis*	MCCC 1A08372	NR_148786.1

**Table 3:** Number of isolates in each group.

	Group 1	Group 2	Group 3	Group 4	Group 5	Group 6	Group 7
From sample 2019.1.30	6	0	0	0	0	0	0
From sample 2021.1.26; Culture at 30 °C	8	3	3	0	0	0	0
From sample 2021.1.26; Culture at 15 °C	9	4	0	0	1	0	0
From sample 2021.1.28; Culture at 30 °C	11	1	3	2	1	1	0
From sample 2021.1.28; Culture at 15 °C	5	2	2	0	2	0	0
From sample 2021.2.9; Culture at 30 °C	6	3	1	4	0	0	0
From sample 2021.2.9; Culture at 15 °C	5	5	2	1	0	0	1

## Data Availability

The data presented in this study are openly available in Supplementary File S1.

## Supplementary Materials

The following are available online at https://www.mdpi.com/article/10.3390/microorganisms9081760/s1, Supplementary File S1. Mega file used in the phylogenetic analysis.
